# MASLD-Related HCC: A Comprehensive Review of the Trends, Pathophysiology, Tumor Microenvironment, Surveillance, and Treatment Options

**DOI:** 10.3390/cimb46060356

**Published:** 2024-06-13

**Authors:** Yuming Shi, Erfan Taherifard, Ali Saeed, Anwaar Saeed

**Affiliations:** 1 Department of Medicine, Division of Hematology & Oncology, University of Pittsburgh Medical Center, Pittsburgh, PA 15232, USA; shiy6@upmc.edu (Y.S.); erfantaherifard@gmail.com (E.T.); 2 Department of Medicine, Ochsner Lafayette General Medical Center, Lafayette, LA 70503, USA; asaeedmd@gmail.com; 3 UPMC Hillman Cancer Center, Pittsburgh, PA 15232, USA

**Keywords:** hepatocellular carcinoma, metabolic dysfunction-associated steatotic liver disease, MASLD, nonalcoholic fatty liver disease, NAFLD, population surveillance, hepatectomy, liver transplantation, ablation techniques, tumor microenvironment, immunotherapy, immune checkpoint inhibitors, protein kinase inhibitors

## Abstract

Hepatocellular carcinoma (HCC) represents a significant burden on global healthcare systems due to its considerable incidence and mortality rates. Recent trends indicate an increase in the worldwide incidence of metabolic dysfunction-associated steatotic liver disease (MASLD) and a shift in the etiology of HCC, with MASLD replacing the hepatitis B virus as the primary contributor to new cases of HCC. MASLD-related HCC exhibits distinct characteristics compared to viral HCC, including unique immune cell profiles resulting in an overall more immunosuppressive or exhausted tumor microenvironment. Furthermore, MASLD-related HCC is frequently identified in older age groups and among individuals with cardiometabolic comorbidities. Additionally, a greater percentage of MASLD-related HCC cases occur in noncirrhotic patients compared to those with viral etiologies, hindering early detection. However, the current clinical practice guidelines lack specific recommendations for the screening of HCC in MASLD patients. The evolving landscape of HCC management offers a spectrum of therapeutic options, ranging from surgical interventions and locoregional therapies to systemic treatments, for patients across various stages of the disease. Despite ongoing debates, the current evidence does not support differences in optimal treatment modalities based on etiology. In this study, we aimed to provide a comprehensive overview of the current literature on the trends, characteristics, clinical implications, and treatment modalities for MASLD-related HCC.

## 1. Introduction

Liver cancer ranks as the sixth most prevalent cancer and is responsible for over 800,000 deaths annually, making it the fourth leading cause of cancer-related mortality worldwide [1,2]. Hepatocellular carcinoma (HCC) constitutes the vast majority of primary liver cancer cases worldwide, representing more than 80% [3]. In recent years, there has been a notable shift in the proportion of new cases of HCC, with a larger percentage being attributed to metabolic liver disease rather than cirrhosis from hepatitis B (HBV) or C virus (HCV) infection, making it the most rapidly expanding contributor to HCC, with significant implications for screening and treatment [4]. Metabolic liver disease is a known risk factor for progression to HCC. Formerly known as nonalcoholic fatty liver disease (NAFLD), metabolic dysfunction-associated steatotic liver disease (MASLD) is the updated term describing liver dysfunction observed in patients with metabolic disease, including the majority of patients previously diagnosed with NAFLD [5]. Despite the recent terminology change, many recent publications still use NAFLD to describe metabolic liver disease. Therefore, this review summarizes the current knowledge regarding the pathogenesis, screening, and treatment of NAFLD- and MAFLD-related HCC under the assumption that they represent the same clinically distinct patient population.

MASLD is characterized by the presence of liver disease accompanied by at least one of the five cardiometabolic risk factors, including a high body mass index (BMI), raised blood sugar, elevated blood pressure, hypertriglyceridemia, and low high-density lipoprotein cholesterol levels [5,6]. It encompasses both simple steatosis, which typically follows a more benign course, and metabolic dysfunction-associated steatohepatitis (MASH; formerly nonalcoholic steatohepatitis, NASH), predisposing patients to fibrosis and/or HCC. Despite its significance, MASLD is often underreported, although estimates suggest global prevalence of 25% globally, with slightly lower prevalence of 24% in North America [7,8]. Concurrent with the increase in the prevalence of MASLD in the general population, there has been a notable surge in the rate of MASLD-related HCC over time. A study in the United States of America on 61,868 adult patients who underwent liver transplantation found that the prevalence of MASLD-related HCC rose from 8.3% of all HCC cases in 2002 to 13.5% in 2012. Additionally, there was a 9% annual increase in MASLD-related HCC rate in the Surveillance, Epidemiology, and End Results registries database from 2004 to 2009, and an 8.5-fold increase in MASLD as the etiology of liver transplantation among candidates on the waiting list between 2002 and 2017 [8,9,10]. Epidemiologic models suggest further increases in MASLD-related HCC rates in all countries, with an estimated increase of 130% in the United States from 2016 to 2030 [4].

MASLD-related HCC tends to develop at a lower rate compared to that of viral HCC, suggesting differences in the progression and risk factors between these two etiologies. In a study conducted in the United States, Ascha et al. discovered that, among a group of 195 patients with MASH cirrhosis undergoing transplantation evaluation, the annual occurrence of HCC was 2.6% for those with MASH cirrhosis and 4.0% for those with HCV-related cirrhosis [11]. Another study found a lower rate of HCC development in patients with MASH cirrhosis, with 10 out of 149 MASH patients affected, compared to 25 out of 147 patients with chronic HCV infection, over a 10-year period [12]. Moreover, MASLD-related HCC can develop at an earlier stage of liver disease compared to other etiologies of chronic liver disease. However, it is worth noting that MASH can progress to HCC even in the absence of cirrhosis, although it is significantly more common in individuals with cirrhosis (0–3% over 20 years compared to 2.4–12.8% over 3–7 years) [13,14,15]. Consistent with this, a meta-analysis comprising 18 studies and encompassing the details of 470,404 patients revealed that the rate of HCC in patients with MASLD without cirrhosis was 0.03 per 100 person-years, whereas the rate in those with cirrhosis was significantly higher at 3.78 per 100 person-years [16]. In addition to cirrhosis being a significant factor in HCC development among patients with MASLD, diabetes mellitus stands out as the most important risk factor among the associated metabolic diseases, with a study showing that those with diabetes have a 2.77-fold (95% confidence interval, CI, of 2.03 to 3.77) higher risk of HCC progression compared to those without diabetes [17,18]. Additionally, the HCC incidence in MASH cirrhosis is higher in older patients, men, and those with higher alcohol intake [11,19,20].

Despite being older and having higher rates of serious comorbidities, such as cardiovascular conditions, compared to those with viral HCC, evidence suggests that patients with MASLD-related HCC tend to have better preserved liver function. These factors may contribute to the inconsistent findings regarding the survival outcomes between MASLD-related and viral HCC. In this context, some studies have shown a poorer prognosis in terms of survival in those with MASLD-related HCC [21,22]. In a study involving 1119 patients with HCC, it was revealed that patients with MASLD-related HCC were significantly older and had higher rates of comorbidities and complications, such as diabetes mellitus, myocardial infarction, and cerebrovascular events [21]. Interestingly, despite the comparable rate of cirrhosis between those with and those without MALSD in this patient cohort, the MASLD-related HCC patients had better liver function and lower Model for End-Stage Liver Disease (MELD) scores. The OS was, however, shorter by about 4 months in these patients compared to patients with other etiologies for their HCC. Similarly, a nationwide study in the United States involving 11,522 patients with HCC demonstrated significantly higher mortality rates in those with alcoholic and MASLD-related HCC compared to viral etiologies [23]. The viral-related patients in this study exhibited lower comorbidities and more favorable tumoral characteristics, including lower stages and tumor sizes. This may suggest that the higher mortality rates observed in alcoholic and MASLD-related HCC in some studies may potentially be attributed to the lower likelihood of these patients undergoing HCC screening compared to individuals with other etiologies [24]. Contrary to this, in one study, it was found that, despite patients with MASLD-related HCC being older (median age of 71.3 years vs. 67.1 years) and having their cancers less frequently detected by surveillance, their median OS was similar to that in patients with other etiologies [25]. Additionally, another study found that the differences in survival between MASLD-related HCC patients and HCV-related HCC patients (25.5 months vs. 33.7 months) disappeared after patient matching for age, gender, and tumor characteristics, underscoring the importance of controlling for confounding factors when comparing outcomes between different etiologies [26]. Nonetheless, a recent systematic review and meta-analysis showed that the overall survival (OS) was similar between the two groups (hazard ratio, HR, of 1.05, with 95% CI of 0.92 to 1.20) [27]. These findings highlight the need for further research to elucidate the underlying factors contributing to the survival outcomes between patients with MASLD-related HCC and those with viral HCC. 

In this review, we first offer an overview of the pathogenesis and tumor microenvironment unique to MASLD-related HCC. Following this, we discuss the current clinical recommendations for HCC screening and treatment options, including surgical interventions, locoregional therapies, and systemic therapies. The subsequent section focuses on treating HCC in patients with MASLD-related HCC and the considerations for this patient population. Finally, we address a series of unresolved challenges in MASLD-related HCC and suggest potential directions to enhance patient outcomes.

## 2. Pathogenesis of MASLD-Related HCC and Tumor Microenvironment

**Figure 1:**
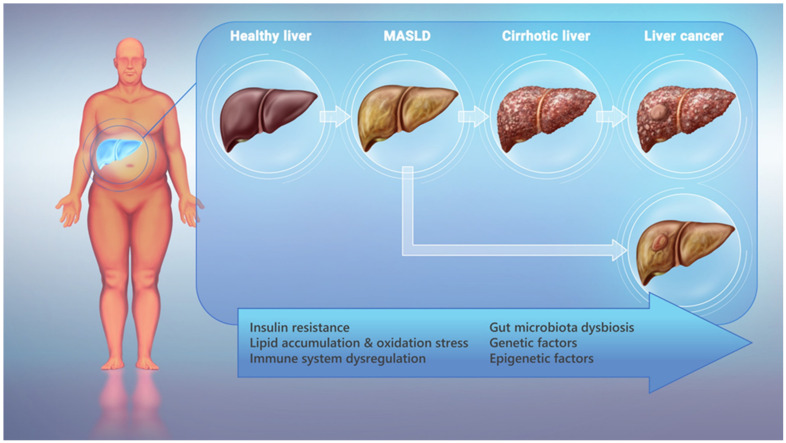
Overview of liver disease progression leading to HCC in patients with MASLD.

While the precise mechanisms underlying the development of HCC in patients with MASLD remain elusive and warrant further investigation, several potential pathways have been proposed. Figure 1 provides an overview of the different stages of liver disease in participants with an unhealthy metabolic status, along with the main mechanisms involved in the progression of HCC among them. The intricate interplay of metabolic irregularities, hyperinsulinemia, insulin resistance, and the accumulation of fatty acids within hepatocytes are believed to contribute to chronic liver inflammation and oxidative stress [28]. The excessive influx of fatty acids overwhelms the hepatocytes’ capacity for management and export, leading to their intracellular accumulation. Consequently, this lipid overload induces stress responses in various cellular compartments, including the endoplasmic reticulum and mitochondria, while also disrupting the Golgi apparatus’ function and protein trafficking through lipotoxicity [29]. Moreover, insulin resistance and the dysregulation of adipokines, as well as the activation of various signaling pathways, such as the PI3K/AKT/mTOR and Wnt/β-catenin pathways, have been implicated in cellular stress, the disruption of balance in the oxidant/antioxidant system, and the inflammatory microenvironment, all promoting hepatocarcinogenesis in the context of MASLD [30,31]. 

Furthermore, emerging evidence underscores the significant role of gut microbiota dysbiosis in MASLD-related HCC [32,33]. Dysbiosis can alter the gut–liver axis and liver metabolism, fostering inflammation and fibrosis, which are pivotal in the progression of liver disease and the onset of HCC [29,30]. In patients with MASLD, studies indicate that there are differences in microbiota composition when compared to patients without MASLD [34,35]. The gut microbiota is vital in maintaining the intestinal barrier’s integrity. Moreover, under normal conditions, the gut microbiota provides crucial nutrients for the growth of the liver cells and participates in the maturation of the immune cells in the gastrointestinal tract, thereby preventing the aberrant inflammation of the hepatic tissue [36]. However, dysbiosis disrupts these functions, leading to increased intestinal permeability, the translocation of bacterial products and toxins into the liver, and the dysregulation of the immune system, collectively exacerbating liver inflammation and damage [37]. Dysbiosis can also result in elevated levels of secondary bile acids, which drive hepatic stellate cells into a state of cellular senescence [38,39]. These senescent stellate cells then produce various growth factors, chemokines, and pro-inflammatory cytokines, contributing to the advancement of liver cancer. In addition to these mechanisms, genetic and epigenetic factors play a crucial role in advancing liver disease toward HCC in MASLD patients [40,41]. These genetic polymorphisms, such as those in the *PNPLA3* gene, and epigenetic modifications, including deoxyribonucleic acid (DNA) methylation, histone acetylation, and the aberrant expression of non-coding ribonucleic acids (RNAs), can contribute to the multifactorial nature of MASLD-associated hepatocarcinogenesis through alterations that affect the hepatic metabolism, inflammation, and cellular transformation processes [42,43]. Despite these insights, a comprehensive understanding of the intricate molecular mechanisms underlying MASLD-related HCC development remains lacking, highlighting the need for further research.

The tumor microenvironment in HCC is characterized by a complex interplay of various immune and non-immune cell populations [44]. The tumor microenvironment plays a crucial role in shaping the progression and prognosis of HCC and its response to treatment. Immune cells, including different subsets of T cells, B cells, natural killer cells, dendritic cells, macrophages, and neutrophils, interact with non-immune stromal cells like cancer-associated fibroblasts and endothelial cells within this microenvironment. Research demonstrates considerable variability in the immune cell compositions among HCC patients, impacting the presence of robust anti-tumoral immune activity and ultimately shaping the disease behavior and prognosis [45,46]. Beyond immune cells, the stromal cells in the tumor microenvironment also play pivotal roles in modulating tumor growth, invasion, and metastasis [47,48]. Notably, studies have elucidated significant differences in the tumor microenvironment immune profile in both terms of innate and adaptive immune cells between viral HCCs and those associated with other etiologies, including MASLD. Understanding the immune landscape and identifying the distinct immune cell compositions and specific immunomodulatory targets within the tumor microenvironment is pivotal in guiding the development of tailored immunotherapeutic strategies for HCC patients with different etiologies. In viral HCC, tumoral lesions tend to be more infiltrated by M0 macrophages, dendritic cells, and mast cells, along with the increased presence of naive B cells, plasma cells, follicular helper T cells, and regulatory T cells, whereas non-viral HCC tends to display a significantly higher infiltration rate of M2 macrophages [49,50]. Consistently with this, an in-depth in vitro study employing cytometry and next-generation sequencing compared the tumor microenvironment in patients with HBV-related HCC and those with non-viral HCC, revealing distinct immune signatures [51]. Specifically, it was revealed that there were higher rates of tissue-resident memory and regulatory T cells in the microenvironment in patients with HBV-related HCC, whereas Tim-3+CD8+ T cells and CD244+ natural killer cells were predominant in non-viral HCC. Transcriptomic analyses further highlighted this disparity between different etiologies, revealing elevated expression levels of *FOXP3* and genes linked to the IL-10 signaling pathway, as well as exhaustion marker genes like *PD-1* and *CTLA4*, in viral HCC, indicating a potentially more immunosuppressive or exhausted microenvironment [51]. Furthermore, the inhibitory impact of PD-1-expressing regulatory T cells on responder T cells was significantly reduced following exposure to PD-1 or PD-L1 inhibitors in samples from patients with HBV-related HCC [51], strengthening the rationale for the use of immunotherapies and suggesting their promising therapeutic benefit for this patient subgroup.

## 3. MASLD-Related HCC Screening

The current clinical practice guidelines do not offer any specific recommendations for HCC screening among individuals who have MASLD. The American Association for the Study of Liver Diseases (AASLD) suggests considering HCC surveillance for patients with cirrhosis of any etiology and those with chronic HBV infection [52]. Consequently, the initial screening protocols used for MASLD-related HCC are identical to those for other etiologies, targeting only MASLD patients who have developed cirrhosis. The European Association for the Study of the Liver (EASL) recommends screening for cirrhotic patients irrespective of the Child–Pugh stage, noncirrhotic patients with HBV and an intermediate or high risk of HCC, and noncirrhotic F3 patients, regardless of etiology [53]. Similarly, the American Gastroenterological Association (AGA) asserts that an MASLD diagnosis alone does not warrant HCC screening, and screening should be considered for MASLD patients with noninvasive markers indicative of advanced fibrosis or cirrhosis [54].

Liver ultrasound is a non-invasive imaging technique commonly used in the diagnosis and surveillance of liver diseases, using sound waves to generate images of the liver and surrounding organs. Despite the common use of ultrasound, it is operator-dependent and its effectiveness in detecting small lesions is not optimal and is variable, with a sensitivity range of 40–80% in the general population [55]. Challenges in its effectiveness and in lesion detection are particularly more pronounced in specific patient populations, including males, individuals with a higher BMI, and those with advanced cirrhosis, as well as in hospitalized patients [56]. Moreover, ultrasound was found to be inadequate in over one third of patients with MASLD-related cirrhosis and had decreased quality and sensitivity in this patient population compared to those with other etiologies, partially attributable to their higher BMIs and the presence of a thicker subcutaneous fat layer in these patients, which weakens the ultrasound beam [56,57]. In addition to imaging techniques, protein biomarkers, particularly alpha-fetoprotein (AFP), are commonly used for diagnosis, with no discernible differences observed between their levels in MASLD-related HCC and other etiologies [58]. AFP, primarily produced by the liver and yolk sac during fetal development, exhibits elevated levels in liver cancer due to various factors, including the release of AFP directly from the cancer cells or by the disruption of normal liver cellular function and, thereby, the leakage of AFP. However, AFP levels cannot be reliably used to detect smaller tumors (<4 cm), with approximately two thirds of such cases presenting with AFP levels below the diagnostic thresholds and approximately 20% of HCCs not producing AFP [27]. The current evidence suggests utilizing a combination of liver ultrasound and AFP for surveillance in high-risk populations, as the diagnostic odds ratio, sensitivity, and cost-effectiveness analyses have demonstrated the superiority of this combination over each modality alone [59,60].

Lectin-bound AFP (AFP-L3) and des-γ-carboxy-prothrombin (DCP or PIVKA-II) are two additional biomarkers currently employed in the surveillance of HCC. During the development of HCC, the carbohydrate chain of AFP undergoes modification by fucosyltransferase, resulting in the formation of AFP-L3. AFP-L3 exhibits sensitivity ranging from 40% to 90% and specificity exceeding 90%. DCP is an immature prothrombin lacking carboxylation at certain glutamate residues, produced due to a post-translational defect in malignant cells. DCP demonstrates sensitivity between 48% and 62% and specificity ranging from 81% to 98%. The combination of AFP-L3 and DCP is routinely used in Japan, where it was observed to offer improved sensitivity and specificity compared to each biomarker individually [61]. Furthermore, combining biomarkers into composite scores, such as GALAD (AFP, AFP-L3, and DCP in addition to age and gender), enhances the diagnostic accuracy. Validated in Germany and the Mayo Clinic, the GALAD score demonstrates high sensitivity (85.6–91%) and specificity (85–93.3%) for HCC detection [62]. GALAD is further improved over ultrasound alone with GALADUS, which incorporates ultrasound findings [63,64,65]. Additionally, the Doylestown algorithm, integrating age, gender, liver enzymes, and AFP, provides another avenue for accurate diagnosis and risk assessment in patients with chronic liver disease [66]. Currently, ongoing investigations are exploring the validation of such combination scores in MASLD-related HCC.

## 4. HCC Treatment

Treatment modalities for HCC encompass a spectrum of options ranging from surgical interventions and locoregional therapies to systemic treatments. Due to the intricate nature of HCC management, these patients must be evaluated and managed within a multidisciplinary care framework. This approach ensures comprehensive assessment by a team of specialists to guide the treatment through the thorough consideration of each patient’s unique clinical profile and has been shown to improve the OS [67,68]. These factors include tumor features such as the size and number, as well as the presence or absence of metastasis, the severity of liver dysfunction and extent of cirrhosis, the patency of the portal vein, operative risks, and the overall patient performance status [69]. Therefore, a meticulous evaluation of the treatment options in the context of comorbidities and anticipated treatment-related toxicities is paramount to optimize the outcomes in these patients.

### 4.1. Surgical Interventions

Surgical resection and liver transplantation are among the primary curative treatments for HCC. Surgical resection involves removing the tumor-containing part of the liver and is suitable for patients without portal hypertension and with localized disease and sufficient liver function [70]. Liver transplantation is preferred for patients with early-stage HCC who are not eligible for resection due to advanced liver disease or unresectable tumors. It offers advantages such as removing both detectable and undetectable tumor lesions, along with the cirrhotic liver, reducing the recurrence risk [71]. However, transplantation is limited by factors like donor availability, the tumor burden, and the patient candidacy criteria, with the Milan criteria used to ensure transplant success by selecting patients with limited tumor burdens [72,73,74]. Currently, efforts are underway to expand these criteria for broader patient inclusion. Additionally, there are strategies for patients exceeding the Milan criteria, including split-liver transplantation, living-donor liver transplantation, or downstaging therapy [75].

### 4.2. Locoregional Options

Local ablative techniques offer alternative curative options for patients with solitary lesions who are not suitable candidates for surgical resection due to factors such as the lesion size or location. These techniques may be considered as the initial treatment, especially for centrally located lesions or early-stage HCCs with small sizes. They can also serve as bridge therapies for patients awaiting liver transplantation or as palliative measures in specific cases [52,76]. Techniques include thermal methods like radiofrequency or microwave ablation, chemical approaches with ethanol or acetic acid injection, cryoablation, or electrical pulse application, with thermal methods being the most commonly used [75,77]. Larger lesions or those close to critical structures may benefit from other locoregional ablative strategies, such as external beam radiation therapy [76]. Additionally, arterially directed therapies represent another treatment modality for HCC among locoregional options. These interventions, administered via the arteries, encompass transarterial embolization (TAE), transarterial chemoembolization (TACE), and transarterial radioembolization (TARE) and are primary treatments for intermediate-stage and unresectable HCC [52,78,79]. They not only aim to diminish the blood flow to the tumor, inducing necrosis, but also to deliver chemotherapeutic agents or radiation directly to the tumor [80].

### 4.3. Systemic Therapies

The use of systemic therapies is another therapeutic option for patients with HCC, mainly used for those with advanced-stage HCC. Presently, there are two main categories of approved systemic therapies for advanced HCC patients: immune checkpoint inhibitors (ICIs) and tyrosine kinase inhibitors (TKIs). ICIs target key checkpoints in the immune system, specifically programmed cell death protein 1 (PD-1) and its ligand (PD-L1) and cytotoxic T lymphocyte antigen (CTLA-4), counteracting the immunosuppressive or exhausted microenvironment of HCC and enhancing the immune system’s ability to fight the cancer. TKIs, the other class of drugs used as a systemic therapy, block the actions of specific tyrosine kinases, thereby disrupting signaling pathways responsible for regulating cell growth, division, and angiogenesis. By impeding these pathways, TKIs play a pivotal role in curbing the uncontrolled proliferation of cancer cells, thereby slowing disease progression and improving patient outcomes.

**Figure 2:**
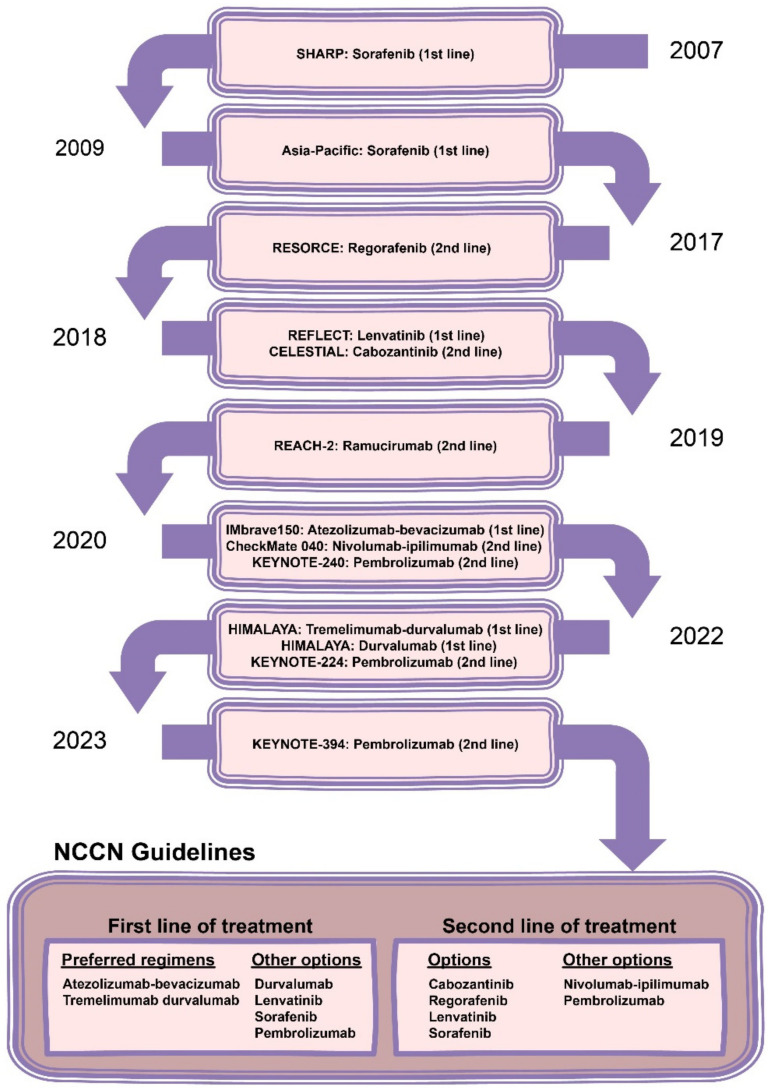
Timeline of the evolution of systemic therapies for patients with advanced HCC and current recommendations in NCCN Guidelines regarding first line and second line of treatment for patients with advanced HCC; order of treatment options does not imply priority.

Until 2017, sorafenib, an antiangiogenic TKI, was the standard of care and the sole systemic therapy option available for patients with advanced HCC. Nevertheless, significant advancements have since been made in the realm of systemic therapies, diversifying the treatment options for patients (Figure 2). Following sorafenib, regorafenib emerged as the subsequent TKI approved for patients with advanced HCC, reserved for those who had progressed on sorafenib, based on conclusive findings from the RESORCE trial [81]. The landscape evolved further with the introduction of other TKIs, including lenvatinib, cabozantinib, and ramucirumab, alongside the advent of ICIs for patients with advanced HCC [81,82,83,84,85]. The introduction of ICIs, notably through trials such as IMbrave150 and HIMALAYA, reshaped the landscape of first-line treatment, establishing combinations like atezolizumab plus bevacizumab and durvalumab plus tremelimumab as the preferred options for this patient population [86,87,88,89,90,91,92,93,94,95,96]. Figure 2 also represents the current first-line and second-line treatment options recommended by the National Comprehensive Cancer Network Clinical Practice Guidelines in Oncology for patients with advanced HCC [69]. 

### 4.4. MASLD-Related HCC Treatment

HCC arising in the context of MAFLD or MASH introduces several distinct challenges and considerations compared to other etiologies of HCC, particularly viral hepatitis. The current clinical practice guidelines have not incorporated the etiology of HCC into the determination of treatment plans for these patients. The inclusion of multidisciplinary care recommendations in these guidelines, however, implicitly acknowledges the importance of considering the etiology of HCC in treatment decisions. Additionally, the prevailing body of evidence upon which these guidelines were developed was derived from studies mainly focusing on patients with viral hepatitis, resulting in a knowledge gap in addressing the distinctive complexities presented by HCC in individuals with MASLD. 

In general, patients with MASLD-related HCC are older, have a poorer overall performance status, and have higher rates of metabolic syndrome-associated comorbidities, such as diabetes mellitus, weight excess, and cardiovascular diseases, compared to patients with other etiologies. These health conditions not only necessitate the execution of specific assessments and management both prior to and following the surgical intervention but also may limit the treatment options in these patients due to unacceptable safety risks [97]. In a comprehensive systematic review and meta-analysis, incorporating data from 61 studies encompassing 94,636 patients diagnosed with HCC, no significant disparities were, however, observed in the likelihood of being assigned to one of the main treatment modalities for HCC—curative therapy, palliative therapy, or best supportive care—between individuals with MASLD and those with HCC stemming from other etiologies [27]. However, intriguingly, the analyses revealed that patients with MASLD-related HCC exhibited lower odds of receiving liver transplants (odds ratio, OR, of 0.49, with 95% CI of 0.29 to 0.84), but higher odds of undergoing surgical resection for the tumor (OR of 1.59 with 95% CI of 1.16–2.18), compared to individuals with HCC from other etiologies. The odds of undergoing ablative procedures were, however, found to be similar across both groups. These findings suggest a complex interplay of factors influencing treatment decisions, including the absence of cirrhosis, among a significantly higher proportion of patients with MASLD-related HCC, advanced age, higher comorbidity rates, and less favorable metabolic profiles, which may contribute to both a reduced necessity for and the feasibility of liver transplantation within this population. Additionally, the analyses highlighted the higher likelihood of uni-nodular lesions among patients with MASLD-related HCC, potentially rendering surgical resection a more suitable option over liver transplantation. 

Despite the marked disparities in the overall health status between patients with MASLD-related HCC and those with other HCC etiologies, research indicates that the survival outcomes are not inferior in MASLD-related HCC patients. The results from a meta-analysis revealed no significant differences in OS between MASLD-related HCC and other etiologies, regardless of the treatment modality [27]; however, MASLD patients demonstrated higher disease-free survival rates in the overall cohort and subgroups of patients who had curative treatment and those who underwent liver resection, but not among those who received liver transplantation. These observations may stem from the notably lower rate of cirrhosis in MASLD patients, as cirrhosis has been shown to be linked to both increased mortality and a higher recurrence risk [98,99]. Notably, two key observations further support this notion: the lack of significant differences in disease-free survival among liver transplant recipients, where the liver is entirely removed and the role of cirrhosis, as a determinant factor is eliminated, and the higher mortality rate associated with MASLD-related HCC compared to other etiologies in a sensitivity analysis on cirrhotic patients. These findings regarding the outcomes of curative treatment in patients with MASLD-related HCC compared to others were also observed in another meta-analysis of this patient population [100]. 

There is a growing body of evidence indicating that the etiology of HCC could affect the response to systemic therapy and ultimately impact patient outcomes. Currently, there are no results from phase III randomized clinical trials (RCTs) regarding the effect of these systemic therapy regimens in patients with MASLD-related HCC. Most of these trials have reported stratified subgroup results for three subgroups: HBV-related, HCV-related, and non-viral HCC. Therefore, the evidence available from these trials regarding the specific effectiveness of systemic therapy for patients with MASLD-related HCC is mainly from the subgroup results of the non-viral group. Table 1 provides an overview of selected phase III randomized pivotal trials investigating the efficacy and safety of various systemic therapy regimens for patients with advanced HCC. Specifically, it includes trials that report results stratified by patient subgroup based on the etiology, with non-viral HCC included among these subgroups. As demonstrated in Table 1, the treatment outcomes can vary significantly depending on the underlying etiology of HCC. It is also noteworthy that, even within the same etiology, there are notable differences from one trial to another, indicating the complexity in assessing the efficacy of systemic therapies based on the etiology of HCC.

Although experimental studies, retrospective reports, early trials, and the findings of two meta-analyses initially suggested the lower efficacy of ICIs in patients with MASLD-related HCC, subsequent studies have yielded contrary results [88,106,107,108,109,110]. In a meta-analysis of data from the pivotal trials of IMbrave150, CheckMate-459, and KEYNOTE-240, it was revealed that, despite the presence of a significant survival benefit in the overall patient cohort treated with ICIs, this benefit was not superior to that of the control group (sorafenib in the first two trials and placebo in the latter) for patients with non-viral HCC [106]. However, notably, patients with HBV-related or HCV-related HCC showed superior outcomes with ICI treatment compared to the control group. Across these three trials included in the meta-analysis, as well as the majority of other investigations on systemic therapy, as mentioned earlier, the patients were typically categorized based on their viral or non-viral etiologies, and the individual response to treatment among MASLD-related HCC cases was not reported. Therefore, the lower efficacy observed in patients with non-viral HCC may not necessarily apply to those with MASLD-related HCC. Supporting this, a post-hoc analysis of the IMbrave150 data showed no significant difference in objective responses, OS, or progression-free survival (PFS) between patients with MASLD and those with other etiologies [108]. Similarly, a meta-analysis of observational and interventional studies on the efficacy and safety of atezolizumab and bevacizumab combination therapy in patients with advanced HCC found comparable radiological responses and PFS in patients with viral and non-viral HCCs [111]. Additionally, the results of the phase III HIMALAYA trial demonstrated that both the durvalumab monotherapy and its combination with tremelimumab, when compared to sorafenib, were associated with significantly longer OS in patients with non-viral etiologies, similar to the overall patient population [88]. A recent meta-analysis, published as a Letter to the Editor on the findings from RCTs, revealed survival benefits for both viral and non-viral HCC, with no significant differences observed between the two groups [110].

The effectiveness of TKIs in patients with non-viral HCC has also yielded heterogeneous findings [107,112,113,114,115,116,117,118,119]. A meta-analysis of data from phase III trials including REACH, REACH-2, CELESTIAL, METIV-HCC, and JET-HCC, encompassing a total of 2083 patients, revealed differing outcomes [107]. While there was a reduction in the risk of mortality for patients with viral HCC (HR of 0.81 with 95% CI of 0.71 to 0.92), there were no significant increases in OS observed for non-viral patients (HR of 0.82 with 95% CI of 0.67 to 1.01). Among the phase III RCTs evaluating the effectiveness of various TKIs, there are trials, e.g., the REACH-2 and the CELESTIAL trials, that have provided data on patients with MASLD [83,84]. However, despite this inclusion, the subgroup analysis based on the etiology in these studies for OS and PFS categorized patients into HCV-related, HBV-related, and non-viral HCCs, thereby limiting the ability to specifically assess the outcomes for MASLD-related HCC patients within the study. In a multicenter retrospective study involving 557 patients with unresectable HCC who underwent treatment with lenvatinib, with up to two thirds receiving it as their first line of treatment, the study found that the PFS, but not OS, was significantly higher in patients with MASLD compared to those with viral or alcohol-related HCC [120]. Similar superior efficacy with lenvatinib was observed in another retrospective study in patients with MASLD [112]. As a result, similar to ICIs, the effectiveness of TKIs in this patient population remains a subject of ongoing debate, highlighting the need for further studies with stratified results on patients with MASLD-related HCC and larger sample sizes in this subgroup to achieve statistical power.

## 5. Current Challenges and Future Directions

The survival outcomes have been observed to be superior in patients with MASLD-related HCC compared to those with other etiologies, despite the presence of larger tumor diameters, older age, and the higher prevalence of cardiometabolic comorbidities among them. This disparity is likely attributed to the higher proportion of non-cirrhotic patients and better liver functionality within the MASLD-related HCC cohort. The observed superior survival outcomes in MASLD-related HCC, despite the lower surveillance rates in this patient population, underscore the potential for even higher rates of outcomes and highlight the importance of prioritizing tailored early detection and surveillance strategies for this particular group. Advances in imaging techniques, biomarker identification, risk stratification methods, and artificial intelligence algorithms present promising avenues for the refinement of screening protocols and the enhanced detection of early-stage disease, thereby facilitating timely intervention and ultimately leading to enhanced survival rates [121,122].

Despite the notable progress in the management of HCC, particularly with the advent of systemic agents showing promising efficacy and safety profiles, several areas require further exploration to tackle existing challenges and enhance patient outcomes. While ICIs and TKIs have demonstrated effectiveness in advanced HCC, there remains controversy regarding the adequacy of the treatment response in certain patient subgroups, notably those with MASLD-related HCC. The current literature often lacks specific analyses focusing on the efficacy of various agents in patients with MASLD-related HCC, a subgroup distinguished by a unique tumor microenvironment. This knowledge gap underscores the necessity for investigations to further evaluate the efficacy of ICIs and TKIs in this patient population. Second, there is a lack of comparative data demonstrating optimal subsequent-line systemic therapy, highlighting the ongoing need for further research efforts in systemic therapies for HCC. Exploring novel drug targets, combination therapies, and treatment sequencing holds promise in enhancing the patient outcomes in HCC management [123].

Additionally, despite improvements in the outcomes of HCC patients, challenges persist regarding suboptimal objective response rates and disease control rates, along with the notable incidence of side effects and the economic burden, posing significant challenges in clinical practice. Identifying responders and non-responders and patient subgroups likely to respond to specific therapies is paramount, necessitating a deeper understanding of the treatment resistance mechanisms, including immune evasion and the tumor microenvironment. The validation of reliable biomarkers holds promise in guiding treatment decisions and prognosis prediction in HCC. As the AFP threshold requirement for ramucirumab is currently the sole approved biomarker, diversifying this biomarker landscape is imperative in optimizing the therapeutic outcomes [124].

Lastly, among the key issues to address is the imperative for further efforts to integrate multidisciplinary care into the management of HCC. The multidisciplinary approach to HCC management has demonstrated significant benefits in optimizing treatment decisions and patient outcomes [67,68,125]. This necessity is particularly pronounced in patients with MASLD-related HCC, given their older age and higher rates of cardiometabolic comorbidities. However, the current utilization of multidisciplinary care remains suboptimal [126], emphasizing the critical need to enhance its implementation to ensure comprehensive and coordinated management for patients with HCC.

## Figures and Tables

**Table 1:** Selected phase III randomized pivotal trials assessing the efficacy and safety of different regimens for systemic therapy in advanced HCC.

Trial Name and Number	Arms of the Study	Number of Participants in Each Arm	Patient Subgroup Based on Etiology	Overall Survival HR	Progression-Free Survival HR
1st line of treatment					
IMbrave150 (NCT03434379) [86]	Atezolizumab-bevacizumab vs. sorafenib	336 (non-viral: 30% vs. 32%)	Overall	0.58 (0.42–0.79)	0.59 (0.47–0.76)
			Hepatitis B	0.51 (0.32–0.81)	0.47 (0.33–0.67)
			Hepatitis C	0.43 (0.22–0.87)	0.69 (0.39–1.20)
			Non-viral	0.91 (0.52–1.60)	0.71 (0.47–1.08)
COSMIC-312 (NCT03755791) [101]	Atezolizumab-bevacizumab vs. sorafenib	649 (non-viral: 39% vs. 40%)	Overall	0.90 (0.69–1.18)	0.63 (0.44–0.91)
			Hepatitis B	0.53 (0.33–0.87)	0.46 (0.29–0.73)
			Hepatitis C	1.10 (0.72–1.68)	0.64 (0.38–1.09)
			Non-viral	1.18 (0.78–1.79)	0.92 (0.60–1.41)
HIMALAYA (NCT03298451) [88]	Tremelimumab-durvalumab vs. sorafenib	782 (non-viral: 41% vs. 43%)	Overall	0.78 (0.65–0.93)	0.90 (0.77–1.05)
			Hepatitis B	0.64 (0.48–0.86)	-
			Hepatitis C	1.06 (0.76–1.49)	-
			Non-viral	0.74 (0.57–0.95)	-
	Durvalumab vs. sorafenib	782 (non-viral: 42% vs. 43%)	Overall	0.86 (0.73–1.03)	1.02 (0.88–1.19)
			Hepatitis B	0.78 (0.58–1.04)	-
			Hepatitis C	1.05 (0.75–1.48)	-
			Non-viral	0.82 (0.64–1.05)	-
CheckMate 459 (NCT02576509) [89]	Nivolumab vs. sorafenib	743 (non-viral: 45% vs. 45%)	Overall	0.85 (0.72–1.02)	0.93 (0.79–1.10)
			Hepatitis B	0.77 (0.56–1.05)	-
			Hepatitis C	0.71 (0.49–1.01)	-
			Non-viral	0.95 (0.74–1.22)	-
RATIONALE-301 (NCT03412773) [102]	Tislelizumab vs. sorafenib	674 (non-viral: 24% vs. 24%)	Overall	0.85 (0.71–1.02)	1.11 (0.92–1.33)
			Hepatitis B	0.91 (0.73–1.14)	-
			Hepatitis C	0.64 (0.38–1.08)	-
			Non-viral	0.78 (0.55–1.12)	-
LEAP-002 (NCT03713593) [103]	Pembrolizumab-lenvatinib vs. lenvatinib	794 (non-viral: 37% vs. 39%)	Overall	0.84 (0.71–1.00)	0.83 (0.71–0.98)
			Hepatitis B	0.75 (0.58–0.97)	-
			Hepatitis C	0.86 (0.60–1.24)	-
			Non-viral	0.84 (0.67–1.05)	-
CARES-310 (NCT03764293) [104]	Camrelizumab-rivoceranib vs. sorafenib	543 (non-viral: 15% vs. 17%)	Overall	0.62 (0.49–0.80)	0.52 (0.41–0.65)
			Hepatitis B	0.66 (0.50–0.87)	0.57 (0.45–0.72)
			Hepatitis C	0.45 (0.18–1.16)	0.46 (0.21–1.05)
			Non-viral	0.71 (0.37–1.36)	0.55 (0.33–0.93)
2nd line of treatment					
KEYNOTE-240 (NCT02702401) [94]	Pembrolizumab vs. placebo	413 (non-viral: 59% vs. 63%)	Overall	0.78 (0.61–1.00)	0.72 (0.57–0.90)
			Hepatitis B	0.57 (0.35–0.94)	0.70 (0.44–1.13)
			Hepatitis C	0.96 (0.48–1.92)	0.46 (0.24–0.90)
			Non-viral	0.88 (0.64–1.20)	0.75 (0.56–1.01)
KEYNOTE-394 (NCT03062358) [95]	Pembrolizumab vs. placebo	453 (non-viral *: 21% vs. 19%)	Overall	0.79 (0.63–0.99)	0.74 (0.60–0.92)
			Hepatitis B	0.78 (0.61–0.99)	0.77 (0.61–0.98)
			Non-viral	0.87 (0.53–1.44)	0.58 (0.36–0.94)
CELESTIAL (NCT01908426) [83]	Cabozantinib vs. placebo	707 (MASLD: 9% vs. 10%; non-viral: 60% vs. 59%)	Overall	0.76 (0.63–0.92)	0.44 (0.36–0.52)
			Hepatitis B	0.69 (0.51–0.94)	0.31 (0.23–0.42)
			Hepatitis C	1.11 (0.72–1.71)	0.61 (0.42–0.88)
			Non-viral	0.72 (0.54–0.96)	0.48 (0.36–0.63)
REACH (NCT01140347) [105]	Ramucirumab vs. placebo	565 (non-viral: 37% vs. 37%)	Overall	0.87 (0.72–1.05)	0.63 (0.52–0.75)
			Hepatitis B	0.79 (0.58–1.07)	0.60 (0.45–0.81)
			Hepatitis C	0.88 (0.61–1.26)	0.61 (0.43–0.86)
			Non-viral	0.95 (0.69–1.30)	0.67 (0.49–0.90)
REACH-2 (NCT02435433) [84]	Ramucirumab vs. placebo	292 (MASLD: 10% vs. 2%; non-viral: 39% vs. 29%)	Overall	0.71 (0.53–0.95)	0.45 (0.34–0·60)
			Hepatitis B	0.84 (0.52–1.3)	0.43 (0.28–0.68)
			Hepatitis C	0.76 (0.43–1.3)	0.33 (0.19–0.60)

* In this study, only approximately 1% of the total population had HCV; hence, we considered those patients without HBV as the non-viral group. Abbreviations: HR, hazard ratio; MASLD; metabolic dysfunction-associated steatotic liver disease.
